# Supplementary material for: Asymmetric activation mechanism of a homodimeric red light-regulated photoreceptor
Source: eLife. 2018 Jun 5;7:e34815. doi: 10.7554/eLife.34815 (PMC6005682; doi:10.7554/eLife.34815)
Supplement: Supplementary file 1. [file elife-34815-supp1.docx]

Supplementary Information for

Asymmetric activation mechanism of a homodimeric red light regulated photoreceptor.

Geoffrey Gourinchas^1^, Udo Heintz^2^ & Andreas Winkler^1,*^.

*For correspondence: [Andreas.Winkler@TUGraz.at](mailto:Andreas.Winkler@TUGraz.at)

**This PDF file includes:**

Supplementary Tables 1 to 3

**Supplementary Table 1. Overview of thermal dark state recoveries for the *Is*PadC constructs used in this study.** * For comparison of individual variants all measurements were done at a protein concentration of 2 μM and initially fitted with the sum of two exponentials. Since we observed suboptimal fits of dark-state recoveries of the variants that feature extremely slow recovery contributions we used the sum of three exponentials to fit the recovery of these variants. For variants featuring excessively long recoveries the fit included a fixed known endpoint (y0), which was obtained from the corresponding dark state spectra. After red light illumination of 1 min, changes in absorption at 710 nm were followed, with automatic sampling every 5 s and an integration time of 0.01 s. The contribution of each phase to the thermal recovery is represented as relative amplitude. The SE of the estimate from the nonlinear curve fit corresponding to y = A1*exp(−x/τ1) + A2*exp(−x/τ2) + y0 for a sum of two exponentials, and to y = A1*exp(−x/τ1) + A2*exp(−x/τ2) + A3*exp(-x/τ3) + y0 for a sum of three exponentials, were used as error indicators. ^#1^ Recovery time constants measured as described above for *Is*PadC at 10 μM. Due to a dependence of the recovery kinetics on protein concentration, *Is*PadC at higher protein concentrations required a fit based on the sum of three exponentials to fit the dark-state recovery. ^#2^ After red light illumination, the 10 μM sample was kept in the dark and aliquots were removed at different time points to follow the changes in the acid denatured spectra of biliverdin linked peptides by diluting the sample 1:10 in methanol/TCA 0.1%.

|  | **Dark state recovery @ 710 nm^*^** | | | | | | | |
| --- | --- | --- | --- | --- | --- | --- | --- | --- |
| **Concentration** | **Construct** | **τ_1_ (s)** | **relative A_1_ (%)** | **τ_2_ (s)** | **relative A_2_ (%)** | **τ_3_ (s)** | **relative A_3_ (%)** |  |
| 2 μM | *Is*PadC | 18.5 ± 0.4 | 34 ± 0.5 | 100.0 ± 0.7 | 66 ± 0.5 | - | - |  |
|  | *Is*PadC^Reg1^ | 18.2 ± 0.3 | 37 ± 0.5 | 141.5 ± 0.8 | 48 ± 0.4 | 6,930 ± 50 | 15 ± 0.5 |  |
|  | *Is*PadC^Reg2^ | 28.5 ± 0.3 | 37 ± 0.9 | 3,480 ± 4 | 63 ± 0.8 | - | - |  |
|  | *Is*PadC^Reg2.a^ | 34.9 ± 0.2 | 36 ± 0.8 | 1,300 ± 20 | 3 ± 0.2 | 270,000 ± 1000 | 61 ± 0.7 |  |
| 10 μM | *Is*PadC^#1^ | 18.2 ± 0.2 | 37 ± 0.4 | 110.7 ± 0.6 | 48 ± 0.4 | 2,550 ± 50 | 15 ± 0.1 |  |
|  | *Is*PadC^#2^ | 10.2 ± 0.8 | 33 ±0.1 | 89 ± 6 | 43 ± 0.1 | 6,500 ± 5,000 | 24 ± 0.4 |  |

**Supplementary Table 2. Crystallographic data collection and refinement statistics.**

|  | *Is*PadC^Reg2^ (PDB:6ET7)* |
| --- | --- |
| **Data collection** |  |
| Wavelength, Ȧ | 1.0089 |
| Space group | P 2_1_ 2_1_ 2_1_ |
| Cell dimensions |  |
| *a*, *b*, *c* (Å) | 49.43, 78.36, 443.56 |
| α, β, γ (°) | 90.00, 90.00, 90.00 |
| Resolution (Å) | 58.77 – 2.85 (2.95 - 2.85)^$^ |
| *R*_meas_ (%) | 22.1 (156.4) |
| *I* / σ*I* | 9.28 (1.55) |
| CC (1/2) | 99.6 (55.6) |
| Completeness (%) | 99.9 (99.8) |
| Redundancy | 10.23 (10.76) |
|  |  |
| **Refinement** |  |
| Resolution (Å) | 58.77 – 2.85 |
| No. reflections | 41,744 |
| *R*_work_ / *R*_free_ | 0.21 / 0.27 |
| No. atoms |  |
| Protein | 10,424 |
| Ligand/ion | 86 / 2 |
| Water | 6 |
| *B*-factors |  |
| Protein | 84.00 |
| Ligand/ion | 58.37 |
| Water | 31.26 |
| R.m.s. deviations |  |
| Bond lengths (Å) | 0.008 |
| Bond angles (°) | 1.141 |

^*^1 crystal has been used for data collection. ^$^Values in parentheses are for highest-resolution shell.

**Supplementary Table 3. Overview of oligonucleotides and buffers. (a)** Oligonucleotides used in this study. **(b)** Buffers used for purification and storage of the different *Is*PadC variants.

| Panel a - oligonucleotides | |
| --- | --- |
| Desired construct | **Oligonucleotide (5‘-3‘)** |
| *Is*PadC  ^Reg1^ | fw: CTGAATCTGCTGAATGATCAGCTGGCAGATCTAAATGAAAATCTGGAAAAACTGG  rv: CTGATCATTCAGCAGATTCAGCTGCATGCTAAGTGCCACAATCAGC |
| *Is*PadC  ^Reg2^ | fw: GAATGATCAGCTGGCAGATGCAAATGAAAATCTGGAAAAACTGGTCAGCTTTGATGATCTG  rv: CATCTGCCAGCTGATCATTCAGCAGATTCAGCTGCATGACATCTGCCACAATC |
| *Is*PadC  ^Reg2.a^ | fw: ATCAGCTGGCAGATGCAGTTGAAAATCTGGAAAAACTGGTCAGC  rv: GCATCTGCCAGCTGATCAACCAGCAGATTCAGCTGCATG |
| Panel b – buffer systems | |
| Use | **Buffer composition** |
| Storage buffer | 10 mM HEPES pH 7, 0.5 M NaCl, 2 mM MgCl_2_ |
|  |  |
| Storage buffer for crystallization | 10 mM HEPES pH 7, 150 mM NaCl, 2 mM MgCl_2_ |
|  |  |
| Lysis buffer | 50 mM HEPES pH 7, 0.5 M NaCl, 2 mM MgCl_2_, 10 mM imidazole, 1 mM EDTA |
| Dialysis buffer | 50 mM HEPES pH 7, 0.5 M NaCl, 2 mM MgCl_2_, 1 mM EDTA, 1 mM DTE |
