# Supplementary material for: Asymmetric activation mechanism of a homodimeric red light-regulated photoreceptor
Source: eLife. 2018 Jun 5;7:e34815. doi: 10.7554/eLife.34815 (PMC6005682; doi:10.7554/eLife.34815)
Supplement: Transparent reporting form [file elife-34815-transrepform.docx]

***eLife’s* transparent reporting form**

We encourage authors to provide detailed information *within their submission* to facilitate the interpretation and replication of experiments. Authors can upload supporting documentation to indicate the use of appropriate reporting guidelines for health-related research (see [EQUATOR Network](http://www.equator-network.org/%20)), life science research (see the [BioSharing Information Resource](https://biosharing.org/)), or the [ARRIVE guidelines](http://www.plosbiology.org/article/info:doi/10.1371/journal.pbio.1000412) for reporting work involving animal research. Where applicable, authors should refer to any relevant reporting standards documents in this form.

If you have any questions, please consult our Journal Policies and/or contact us: [editorial@elifesciences.org](mailto:editorial@elifesciences.org).

**Sample-size estimation**

- You should state whether an appropriate sample size was computed when the study was being designed
- You should state the statistical method of sample size computation and any required assumptions
- If no explicit power analysis was used, you should describe how you decided what sample (replicate) size (number) to use

Please outline where this information can be found within the submission (e.g., sections or figure legends), or explain why this information doesn’t apply to your submission:

The study reports the structural and functional characterization of one specific protein present in one microorganism species. Therefore the study did not require the choice of an extended sample-size.

**Replicates**

- You should report how often each experiment was performed
- You should include a definition of biological versus technical replication
- The data obtained should be provided and sufficient information should be provided to indicate the number of independent biological and/or technical replicates
- High-throughput sequence data should be uploaded before submission, with a private link for reviewers provided (these are available from both GEO and ArrayExpress)

Please outline where this information can be found within the submission (e.g., sections or figure legends), or explain why this information doesn’t apply to your submission:

To analyze the initial velocity rate of the various protein variants used in the study triplicate measurements were performed. Therefore each reaction was pipetted separately using the same purified protein batch. Moreover triplicate measurements were also performed during the light fluence measurements. In this case technical replicates were prepared by step-wise illumination of the protein at various photon flux densities. After full saturation the sample was allowed to recover to the ground state and the experiment repeated twice. All explanation about the statistical analysis of the replicate can be found in the material and methods section of the manuscript.

**Statistical reporting**

- Statistical analysis methods should be described and justified
- Raw data should be presented in figures whenever informative to do so (typically when N per group is less than 10)
- For each experiment, you should identify the statistical tests used, exact values of N, definitions of center, methods of multiple test correction, and dispersion and precision measures (e.g., mean, median, SD, SEM, confidence intervals; and, for the major substantive results, a measure of effect size (e.g., Pearson's r, Cohen's d)
- Report exact p-values wherever possible alongside the summary statistics and 95% confidence intervals. These should be reported for all key questions and not only when the p-value is less than 0.05.

Please outline where this information can be found within the submission (e.g., sections or figure legends), or explain why this information doesn’t apply to your submission:

Statistical analysis of the initial velocities, light fluence measurements and hydrogen-deuterium exchange experiments were performed using an N of 3. The sample SD (s) was calculated based on the following formula:

In the case of the kinetic characterization, the sample standard deviation of each time point was used for weighting the linear regression that allowed estimation of the initial velocity. The error indicator in Table 1 reports the error estimate of the linear regression.

The exponential decay of the light-activated proteins thermal reversion was fitted using the formula provided in Supplementary Table 1 of the supplementary information file. The error indicator reported in this case is the standard error of the estimate from the fit. No technical or biological replicates were found necessary in this case.

(For large datasets, or papers with a very large number of statistical tests, you may upload a single table file with tests, Ns, etc., with reference to sections in the manuscript.)

**Group allocation**

- Indicate how samples were allocated into experimental groups (in the case of clinical studies, please specify allocation to treatment method); if randomization was used, please also state if restricted randomization was applied
- Indicate if masking was used during group allocation, data collection and/or data analysis

Please outline where this information can be found within the submission (e.g., sections or figure legends), or explain why this information doesn’t apply to your submission:

As our study does not apply to the observation of population with various genetic backgrounds we do not have samples allocated into groups.

**Additional data files (“source data”)**

- We encourage you to upload relevant additional data files, such as numerical data that are represented as a graph in a figure, or as a summary table
- Where provided, these should be in the most useful format, and they can be uploaded as “Source data” files linked to a main figure or table
- Include model definition files including the full list of parameters used
- Include code used for data analysis (e.g., R, MatLab)
- Avoid stating that data files are “available upon request”

Please indicate the figures or tables for which source data files have been provided:

Figure 2 and 3 – the PDB files have been deposited under accession numbers 6ET7 and 5LLW.
